# Antibiotic exposure is associated with decreased risk of psychiatric disorders

**DOI:** 10.3389/fphar.2023.1290052

**Published:** 2024-01-08

**Authors:** Ilan A. Kerman, Matthew E. Glover, Yezhe Lin, Jennifer L. West, Alexandra L. Hanlon, Anita S. Kablinger, Sarah M. Clinton

**Affiliations:** ^1^ Mental Health Integrated Care Community, Minneapolis Veterans Administration Health Care System, Minneapolis, MN, United States; ^2^ School of Neuroscience, Virginia Tech, Blacksburg, VA, United States; ^3^ Department of Psychiatry and Behavioral Medicine, Virginia Tech Carillion School of Medicine, Roanoke, VA, United States; ^4^ Clinical Research Center for Mental Disorders, Chinese-German Institute of Mental Health, Shanghai Pudong New Area Mental Health Center, Tongji University, Shanghai, China; ^5^ Department of Psychiatry, Shanghai East Hospital, School of Medicine, Tongji University, Shanghai, China; ^6^ Department of Statistics and Center for Biostatistics and Health Data Science, Virginia Tech, Blacksburg, VA, United States

**Keywords:** risk, antibiotic, psychosis, retrospective, suicidality, mood disorders, anxiety, sex

## Abstract

**Objective:** This study sought to investigate the relationship between antibiotic exposure and subsequent risk of psychiatric disorders.

**Methods:** This retrospective cohort study used a national database of 69 million patients from 54 large healthcare organizations. We identified a cohort of 20,214 (42.5% male; 57.9 ± 15.1 years old [mean ± SD]) adults without prior neuropsychiatric diagnoses who received antibiotics during hospitalization. Matched controls included 41,555 (39.6% male; 57.3 ± 15.5 years old) hospitalized adults without antibiotic exposure. The two cohorts were balanced for potential confounders, including demographics and variables with potential to affect: the microbiome, mental health, medical comorbidity, and overall health status. Data were stratified by age and by sex, and outcome measures were assessed starting 6 months after hospital discharge.

**Results:** Antibiotic exposure was consistently associated with a significant decrease in the risk of novel mood disorders and anxiety and stressor-related disorders in: men (mood (OR 0.84, 95% CI 0.77, 0.91), anxiety (OR 0.88, 95% CI 0.82, 0.95), women (mood (OR 0.94, 95% CI 0.89,1.00), anxiety (OR 0.93, 95% CI 0.88, 0.98), those who are 26–49 years old (mood (OR 0.87, 95% CI 0.80, 0.94), anxiety (OR 0.90, 95% CI 0.84, 0.97)), and in those ≥50 years old (mood (OR 0.91, 95% CI 0.86, 0.97), anxiety (OR 0.92, 95% CI 0.87, 0.97). Risk of intentional harm and suicidality was decreased in men (OR 0.73, 95% CI 0.55, 0.98) and in those ≥50 years old (OR 0.67, 95% CI 0.49, 0.92). Risk of psychotic disorders was also decreased in subjects ≥50 years old (OR 0.83, 95 CI: 0.69, 0.99).

**Conclusion:** Use of antibiotics in the inpatient setting is associated with protective effects against multiple psychiatric outcomes in an age- and sex-dependent manner.

## Introduction

Mental illness affects one in five adults in the United States and is one of the leading causes of disability worldwide (Smith, 2014; Bose et al., 2018). Our understanding of the myriad factors that contribute to the etiology of psychiatric disorders remains limited. Antibiotic medications treat bacterial disease by destroying select bacteria throughout the body, which can disrupt the gut microbiome and inflammatory signaling throughout the body (Bercik and Collins, 2014; Zareifopoulos and Panayiotakopoulos, 2017; Palleja et al., 2018). Such alterations may alter the risk of the emergence of a variety of psychiatric conditions (Yuan et al., 2019; Borkent et al., 2022).

Emerging evidence has linked bacterial infections and antibiotic exposure with both susceptibility and with resilience to mental health disorders (Tome and Filipe, 2011; Lambricht et al., 2017; Kridin and Ludwig, 2023). For example, isoniazid, an antibiotic used to treat tuberculosis, inhibits monoamine oxidase activity, thus increasing monoamine levels and in this way producing antidepressant effects (Butler et al., 2019). Other examples include minocycline, which can be an effective adjunct treatment for major depressive disorder (Miyaoka et al., 2012; Zazula et al., 2021). Studies in rodents have documented that presence of specific bacterial species decrease depressive-like behaviors (Lowry et al., 2007; Siebler et al., 2018), while antibiotic administration during adolescence leads to increased anxiety-like behaviors (Lach et al., 2020). In humans, antibiotic exposure during early development can alter neurocognitive function (Slykerman et al., 2019), and increase risk of psychiatric disorders, including ADHD (Rees, 2014; Aversa et al., 2021). Similarly, a retrospective study using a large database of electronic health records from the UK found increased risk of depression and anxiety in adults after antibiotic exposure (Lurie et al., 2015). However, more recent large-scale analyses found no adverse neuropsychiatric outcomes due to antibiotic exposure (Wilcox et al., 2020), and a protective effect for opioid use disorder (Freedman et al., 2022). Neuropsychiatric effects of antibiotic exposure likely depend on antibiotic class, timing of antibiotic administration, and co-administration of other medications (Freedman et al., 2022; Clegg et al., 2023; Kridin and Ludwig, 2023).

To help define effects of antibiotic exposure on mental health we conducted a retrospective cohort study using a large national database from TriNetX (https://www.trinetx.com/). This database contains millions of de-identified, anonymized patient electronic medical records, which we used to determine the risk for major classes of psychiatric diagnoses following antibiotic treatment. Diagnostic classes included: mood disorders, anxiety and stressor-related disorders, intentional self-harm and suicidality, and psychotic disorders. Covariates included twenty-six variables, including demographic variables, comorbid conditions (e.g. pain, inflammation, obesity, etc.), CNS medications and others. Our results indicate that antibiotic administration during hospitalization decreases the risk of all four of the psychiatric outcomes that we examined in a sex- and age-dependent manner.

### Methods

This is an observational retrospective study using weighted data. We utilized data from TriNetX (https://www.trinetx.com/), a global health research network providing access to statistics on electronic medical records (diagnoses, procedures, medications, laboratory values, genomic information) from approximately 69 million patients in 54 large healthcare organizations. TriNetX received a waiver from Western Institutional Review Board (Puyallup, WA) since only aggregated counts, statistical summaries of de-identified information, but no protected health information is received, and no study-specific activities are performed in retrospective analyses. We also received an Institutional Review Board (IRB) waiver locally from the Carilion Clinic IRB.

### Setting

Patient cohorts were identified and analyzed in the TriNetX database in November 2020. All health care organizations available in the database at that time were used in this study. Cases were admitted to the hospital and treated with one or more antibiotics between 2013–2015. Control subjects were hospital inpatients during this same timeframe but did not receive antibiotic treatment. Six months after hospital discharge, we looked for the presence of electronic medical record codes related to mental illness diagnoses. The time gap between the antibiotic exposure window and outcome measures was included in the study design to reduce protopathic bias (Faillie J. L., 2015).

### Participants

Patients were males and females between the ages of 18–89 years old at the start of the Index Event (hospital admission). All patients in the electronic patient records database were included if they were: a) 18 years of age or older; b) admitted inpatients during 2013–2015; c) not receiving antibiotics on admission (or within 6 months prior to admission); d) had no previous record of neuropsychiatric disorders; e) never had documented CRP measures greater than 3 mg/L; and f) had at least one post-discharge follow-up visit recorded in the database at least 6 months after discharge.

*Exclusion Criteria*. Patients in the electronic records database were excluded from our analyses if they were: a) under 18 years of age or older than 89 years of age; b) not admitted inpatients during 2013–2015; c) receiving antibiotics on admission (or within 6 months prior to admission); d) had previous record of neuropsychiatric disorders; e) had documented CRP measures greater than 3 mg/L at any time; or f) did not have at least one post-discharge follow-up visit recorded in the database at least 6 months after discharge.

### Bias

To minimize bias, we followed the Strengthening the Reporting of Observational Studies in Epidemiology (STROBE) reporting guideline (see Supplemental Table S8 for STROBE checklist) (von Elm et al., 2007a; von Elm et al., 2007b; von Elm et al., 2007c; von Elm et al., 2007d). Furthermore, to account for protopathic bias, outcomes were not assessed until 6 months after hospital discharge (Faillie J.-L., 2015). This was done to minimize the association of the outcome with the initial patient complaint during the start of the Index Event. Referral bias was accounted for by sampling across multiple health care organizations. To help account for Berkson bias, where patients with more than one disease are more likely to be hospitalized thus increasing the potential for overestimation in the cases, cohorts were balanced on factors influencing contact with healthcare services (Sutton-Tyrrell, 1991).

### Variables

Outcome measures were assessed 6 months to 5 years after discharge. Primary outcome measures included: 1) anxiety and stress-related disorders, 2) mood disorders, 3) psychotic disorders, and 4) intentional self-harm and suicidality. Supplemental Table S1 provides a list of diagnostic codes for each of these outcomes. The predictor of interest is an antibiotic prescription occurring during inpatient hospitalization. For a full listing of electronic health record codes used to define antibiotic prescription see Supplemental Table S2. Two stratifying variables were considered: sex (male and female) and age group (Age: 18 to 25, Age: 26 to 49, Age: 50 or older). Twenty-six variables were used for weighting variables, including five demographic variables–sex, age at hospitalization, race, ethnicity, and length of hospitalization (Supplemental Table S3), and twenty-one diagnostic and medication variables listed in Supplemental Table S4. In models considering the effect of treatment group by sex, age was included as a continuous weighting variable; in models considering treatment effects by age group, sex was included as a weighting variable. For a full description of the diagnostic variables, including TriNetX codes and the codes that were grouped together, see Supplemental Tables S5–S7. Data for *Antibiotics After Discharge* were collected from 2 weeks after discharge and up to 3 months after discharge. All other characteristics were taken from the beginning of the electronic health record up until 2 weeks post-discharge.

### Study size

The downloaded dataset contained electronic health records from 502,444 adult patients with an index medical encounter between 2013–2015, excluding those with any neuropsychiatric diagnoses prior to the index encounter. The downloaded dataset was further restricted to: a) exclude those having CRP measures >3 mg/L; b) include only index encounters coded as an inpatient hospitalization; and c) include only those with a subsequent medical encounter recorded at least 6 months post-discharge.

### Statistical methods

*Preliminary Analyses*. Derived data fields were calculated for age at hospital admission, length of hospitalization, and antibiotics prescribed post-discharge. In the event of missing data, the underlying mechanism of missingness was evaluated prior to implementing methodology to minimize bias from missing data for matching variables. Age at hospital admission could not be calculated for 2.93% of the sample due to missing year of birth. Age was imputed from the mean in these cases. Sex was missing for one individual and was imputed from the mode. For 2% of the sample, hospital discharge was coded before hospital admission. For these cases, length of hospitalization was set to zero.

*Entropy Balancing*. Entropy balancing uses an algorithm to find a single multiplier for each observation such that all covariates are balanced (Hainmueller, 2012). Entropy balancing was applied to each subset of the data by stratifying variables: female patients, male patients, patients aged 18 to 25, patients aged 26 to 49, and those 50 and older. Patient characteristics included in entropy balancing are shown in Table 1 and Table 2. Demographic patient characteristics included: age at admission, gender, race, and ethnicity. Clinical patient characteristics included medical diagnoses and medications.

**TABLE 1:** Demographics and covariates used in the model for the data stratified by sex. Age at hospitalization and length of hospitalization are shown as mean ± SD. Length of hospitalization is shown in days for informational purposes. For the purposes of entropy balancing and creation of weighted data subsets it was converted to years. Total number of subjects (and their proportion of total cohort) is shown for each category. Absolute standardized mean differences (ASMD) are presented before and after propensity score matching. Abbreviations: NA–not applicable, MSK–musculoskeletal, CNS–central nervous system.

Tables	Exposed	Unexposed	Unadjusted ASMD	Weighted ASMD
**Females (N = 36,718)**				
Total, No.	11,620	25,098	NA	NA
Age at hospitalization (years)	52.45 ± 17.4	53.98 ± 16.76	0.0877	<0.0001
Length of hospitalization (days)	5.56 ± 26.7	3.93 ± 18.19	0.061	<0.0001
**Race**				
American Indian or Alaskan Native	106 (0.91%)	229 (0.91%)	0.0055	<0.0001
Asian	221 (1.90%)	477 (1.90%)	0.0014	<0.0001
African American	1,481 (12.75%)	3,199 (12.75%)	0.0746	<0.0001
Native Hawaiian or Other	12 (0.10%)	26 (0.10%)	0.0002	<0.0001
White	8,342 (71.79%)	18,018 (71.79%)	0.0423	<0.0001
Unknown	1,458 (12.55%)	3,149 (12.55%)	0.0253	<0.0001
**Ethnicity**				
Hispanic or Latino	1,107 (9.53%)	2,391 (9.53%)	0.0396	<0.0001
Not Hispanic or Latino	8,246 (70.96%)	17,811 (70.96%)	0.0261	<0.0001
Unknown	2,267 (19.51%)	4,896 (19.51%)	0.0135	<0.0001
Antibiotics used after discharge	1,899 (16.34%)	4,102 (16.34%)	0.0768	<0.0001
Nicotine use	913 (7.86%)	1,972 (7.86%)	0.0157	<0.0001
Risk Factors	191 (1.64%)	413 (1.64%)	0.0008	<0.0001
CNS Medications	9,581 (82.45%)	20,694 (82.45%)	0.4331	<0.0001
Hormones/Synthetics/Modifiers	7,873 (67.75%)	17,005 (67.75%)	0.3635	<0.0001
Diabetes	573 (4.93%)	1,238 (4.93%)	0.0028	<0.0001
Thyroid/Endocrine Issues	112 (0.96%)	242 (0.96%)	0.002	<0.0001
Obesity	537 (4.62%)	1,160 (4.62%)	0.0134	<0.0001
Other Metabolic Disorders	2,174 (18.71%)	4,696 (18.71%)	0.0518	<0.0001
Diseases of the Heart/Vasculature	1,811 (15.59%)	3,912 (15.59%)	0.0052	<0.0001
Respiratory Diseases/Illnesses	1,158 (9.97%)	2,501 (9.97%)	0.0183	<0.0001
MSK/Joint/Connective Tissue Diseases	1,814 (15.61%)	3,918 (15.61%)	0.0158	<0.0001
Other CNS-Related Issues	584 (5.03%)	1,261 (5.03%)	0.0022	<0.0001
Inflammation/Infection	6,868 (59.10%)	14,834 (59.10%)	0.3227	<0.0001
Pain	10,794 (92.89%)	23,314 (92.89%)	0.4472	<0.0001
Lack of Normal Physiological Development	11 (0.09%)	24 (0.09%)	0.0006	<0.0001
Malnutrition and Nutritional Deficiencies	109 (0.94%)	235 (0.94%)	0.0037	<0.0001
Factors Influencing Mental Health Status	493 (4.24%)	1,065 (4.24%)	0.0113	<0.0001
Healthcare Encounters	2,244 (19.31%)	4,847 (19.31%)	0.0318	<0.0001
Lifestyle/Environmental Stressors	151 (1.30%)	326 (1.30%)	0.0043	<0.0001
Allergy to Antibiotic/Anti-infective Agents	118 (1.02%)	255 (1.02%)	0.0074	<0.0001

	Exposed	Unexposed	Unadjusted ASMD	Weighted ASMD
**Males (N = 25,051)**				
Total, No.	8,594	16,457	NA	NA
Age at hospitalization (years)	57.87 ± 15.02	57.28 ± 15.46	0.0392	<0.0001
Length of hospitalization (days)	6.45 ± 28.13	4.92 ± 26.24	0.0543	<0.0001
**Race**				
American Indian or Alaskan Native	82 (0.95%)	157 (0.95%)	0.0051	<0.0001
Asian	158 (1.84%)	303 (1.84%)	0.0027	<0.0001
African American	817 (9.51%)	1,565 (9.51%)	0.0561	<0.0001
Native Hawaiian or Other	18 (0.21%)	34 (0.21%)	0.0011	<0.0001
White	6,507 (75.72%)	12,461 (75.72%)	0.03	<0.0001
Unknown	1,012 (11.78%)	1,938 (11.78%)	0.0172	<0.0001
**Ethnicity**				
Hispanic or Latino	809 (9.41%)	1,549 (9.41%)	0.0424	<0.0001
Not Hispanic or Latino	5,915 (68.83%)	11,327 (68.83%)	0.0095	<0.0001
Unknown	1,870 (21.76%)	3,581 (21.76%)	0.0329	<0.0001
Antibiotics used after discharge	1,659 (19.30%)	3,177 (19.30%)	0.1131	<0.0001
Nicotine use	1,112 (12.94%)	2,129 (12.94%)	0.022	<0.0001
Risk Factors	183 (2.13%)	350 (2.13%)	0.0031	<0.0001
CNS Medications	7,040 (81.92%)	13,481 (81.92%)	0.4342	<0.0001
Hormones/Synthetics/Modifiers	5,333 (62.05%)	10,212 (62.05%)	0.3442	<0.0001
Diabetes	686 (7.98%)	1,314 (7.98%)	0.0106	<0.0001
Thyroid/Endocrine Issues	88 (1.02%)	169 (1.02%)	0.0007	<0.0001
Obesity	401 (4.67%)	768 (4.67%)	0.0237	<0.0001
Other Metabolic Disorders	2,301 (26.77%)	4,406 (26.77%)	0.0835	<0.0001
Diseases of the Heart/Vasculature	1,817 (21.14%)	3,479 (21.14%)	0.0028	<0.0001
Respiratory Diseases/Illnesses	846 (9.84%)	1,620 (9.84%)	0.0303	<0.0001
MSK/Joint/Connective Tissue Diseases	1,516 (17.64%)	2,903 (17.64%)	0.0509	<0.0001
Other CNS-Related Issues	376 (4.38%)	720 (4.38%)	0.0016	<0.0001
Inflammation/Infection	4,755 (55.33%)	9,105 (55.33%)	0.3179	<0.0001
Pain	8,084 (94.07%)	15,480 (94.06%)	0.4226	<0.0001
Lack of Normal Physiological Development	6 (0.07%)	11 (0.07%)	0.0004	<0.0001
Malnutrition and Nutritional Deficiencies	79 (0.92%)	151 (0.92%)	0.0001	<0.0001
Factors Influencing Mental Health Status	530 (6.17%)	1,015 (6.17%)	0.0247	<0.0001
Healthcare Encounters	1,510 (17.57%)	2,892 (17.57%)	0.0033	<0.0001
Lifestyle/Environmental Stressors	131 (1.52%)	251 (1.52%)	0.0021	<0.0001
Allergy to Antibiotic/Anti-infective Agents	71 (0.83%)	136 (0.83%)	0.0066	<0.0001

**TABLE 2:** Demographics and covariates used in the model for the data stratified by age. Age at hospitalization and length of hospitalization are shown as mean ± SD. Length of hospitalization is shown in days for informational purposes. For the purposes of entropy balancing and creation of weighted data subsets it was converted to years. Total number of subjects (and their proportion of total cohort) is shown for each category. Absolute standardized mean differences (ASMD) are presented before and after propensity score matching. Abbreviations: NA–not applicable, MSK–musculoskeletal, CNS–central nervous system.

	Exposed	Unexposed	Unadjusted ASMD	Weighted ASMD
**18–25 years (N = 3,135)**				
Total, No.	1,052	2,083	NA	NA
Female	755 (71.77%)	1,495 (71.77%)	0.027	<0.0001
Age at hospitalization (years)	22.30 ± 2.04	22.21 ± 1.99	0.046	<0.0001
Length of hospitalization (days)	4.56 ± 9.06	3.12 ± 11.48	0.046	<0.0001
**Race**				
American Indian or Alaskan Native	15 (1.43%)	30 (1.43%)	0.0095	<0.0001
Asian	19 (1.81%)	38 (1.81%)	0.0013	<0.0001
African American	170 (16.16%)	337 (16.16%)	0.029	<0.0001
Native Hawaiian or Other	1 (0.10%)	2 (0.10%)	0.0005	<0.0001
White	634 (60.27%)	1,255 (60.27%)	0.02	<0.0001
Unknown	213 (20.25%)	422 (20.25%)	0.0378	<0.0001
**Ethnicity**				
Hispanic or Latino	158 (15.02%)	313 (15.02%)	0.0691	<0.0001
Not Hispanic or Latino	648 (61.60%)	1,283 (61.60%)	0.0499	<0.0001
Unknown	246 (23.38%)	487 (23.38%)	0.0192	<0.0001
Antibiotics used after discharge	178 (16.92%)	352 (16.92%)	0.0799	<0.0001
Nicotine use	95 (9.03%)	188 (9.03%)	0.0125	<0.0001
Risk Factors	16 (1.52%)	32 (1.52%)	0.0016	<0.0001
CNS Medications	782 (74.33%)	1,548 (74.33%)	0.3223	<0.0001
Hormones/Synthetics/Modifiers	655 (62.26%)	1,297 (62.26%)	0.3048	<0.0001
Diabetes	8 (0.76%)	16 (0.76%)	0.0009	<0.0001
Thyroid/Endocrine Issues	6 (0.57%)	12 (0.57%)	0.0014	<0.0001
Obesity	23 (2.19%)	46 (2.19%)	0.0089	<0.0001
Other Metabolic Disorders	108 (10.27%)	214 (10.27%)	0.0527	<0.0001
Diseases of the Heart/Vasculature	30 (2.85%)	59 (2.85%)	0.0031	<0.0001
Respiratory Diseases/Illnesses	75 (7.13%)	149 (7.13%)	0.029	<0.0001
MSK/Joint/Connective Tissue Diseases	48 (4.56%)	95 (4.56%)	0.0024	<0.0001
Other CNS-Related Issues	33 (3.14%)	65 (3.14%)	0.0046	<0.0001
Inflammation/Infection	535 (50.86%)	1,059 (50.85%)	0.2546	<0.0001
Pain	955 (90.78%)	1,891 (90.78%)	0.3533	<0.0001
Lack of Normal Physiological Development	4 (0.38%)	8 (0.38%)	0.0028	<0.0001
Malnutrition and Nutritional Deficiencies	15 (1.43%)	30 (1.43%)	0.0027	<0.0001
Factors Influencing Mental Health Status	19 (1.81%)	38 (1.81%)	0.0046	<0.0001
Healthcare Encounters	157 (14.92%)	311 (14.92%)	0.0071	<0.0001
Lifestyle/Environmental Stressors	3 (0.29%)	6 (0.29%)	0.0067	<0.0001
Allergy to Antibiotic/Anti-infective Agents	8 (0.76%)	16 (0.76%)	0.0047	<0.0001

	Exposed	Unexposed	Unadjusted ASMD	Weighted ASMD
**26–49 years (N = 17,740)**				
Total, No.	5,973	11,767	NA	NA
Female	4,091 (68.49%)	8,060 (68.49%)	0.016	<0.0001
Age at hospitalization (years)	37.91 ± 6.92	38.69 ± 6.97	0.112	<0.0001
Length of hospitalization (days)	5.56 ± 28.22	4.24 ± 18.48	0.1597	<0.0001
**Race**				
American Indian or Alaskan Native	79 (1.32%)	156 (1.32%)	0.0071	<0.0001
Asian	130 (2.18%)	256 (2.18%)	0.002	<0.0001
African American	844 (14.13%)	1,663 (14.14%)	0.0629	<0.0001
Native Hawaiian or Other	11 (0.18%)	22 (0.18%)	0.0001	<0.0001
White	4,109 (68.79%)	8,094 (68.79%)	0.0225	<0.0001
Unknown	800 (13.39%)	1,576 (13.39%)	0.0314	<0.0001
**Ethnicity**				
Hispanic or Latino	756 (12.66%)	1,489 (12.66%)	0.0525	<0.0001
Not Hispanic or Latino	4,106 (68.74%)	8,089 (68.74%)	0.0434	<0.0001
Unknown	1,111 (18.60%)	2,189 (18.60%)	0.0091	<0.0001
Antibiotics used after discharge	940 (15.74%)	1,852 (15.74%)	0.0715	<0.0001
Nicotine use	704 (11.79%)	1,387 (11.79%)	0.0193	<0.0001
Risk Factors	134 (2.24%)	264 (2.24%)	0.0014	<0.0001
CNS Medications	4,709 (78.84%)	9,277 (78.84%)	0.3804	<0.0001
Hormones/Synthetics/Modifiers	3,650 (61.11%)	7,191 (61.11%)	0.3251	<0.0001
Diabetes	199 (3.33%)	392 (3.33%)	0.0105	<0.0001
Thyroid/Endocrine Issues	68 (1.14%)	135 (1.14%)	0.0037	<0.0001
Obesity	281 (4.70%)	554 (4.70%)	0.0158	<0.0001
Other Metabolic Disorders	763 (12.77%)	1,503 (12.77%)	0.0475	<0.0001
Diseases of the Heart/Vasculature	518 (8.67%)	1,021 (8.68%)	0.0076	<0.0001
Respiratory Diseases/Illnesses	524 (8.77%)	1,032 (8.77%)	0.0215	<0.0001
MSK/Joint/Connective Tissue Diseases	536 (8.97%)	1,056 (8.97%)	0.002	<0.0001
Other CNS-Related Issues	264 (4.42%)	520 (4.42%)	0.0009	<0.0001
Inflammation/Infection	3,290 (55.08%)	6,482 (55.08%)	0.2847	<0.0001
Pain	5,556 (93.02%)	10,945 (93.01%)	0.4094	<0.0001
Lack of Normal Physiological Development	5 (0.08%)	11 (0.09%)	0.0008	<0.0001
Malnutrition and Nutritional Deficiencies	46 (0.77%)	91 (0.77%)	0.0022	<0.0001
Factors Influencing Mental Health Status	140 (2.34%)	276 (2.34%)	0.0045	<0.0001
Healthcare Encounters	1,043 (17.46%)	2,055 (17.46%)	0.0225	<0.0001
Lifestyle/Environmental Stressors	73 (1.22%)	144 (1.22%)	0.0045	<0.0001
Allergy to Antibiotic/Anti-infective Agents	42 (0.70%)	83 (0.70%)	0.0047	<0.0001

	Exposed	Unexposed	Unadjusted ASMD	Weighted ASMD
**≥50 years (N = 40,894)**				
Total, No.	13,189	27,705	NA	NA
Female	6,774 (51.36%)	14,230 (51.36%)	0.0561	<0.0001
Age at hospitalization (years)	64.97 ± 9.26	64.82 ± 9.44	0.0159	<0.0001
Length of hospitalization (days)	6.22 ± 27.87	4.45 ± 23.54	0.0635	<0.0001
**Race**				
American Indian or Alaskan Native	94 (0.71%)	197 (0.71%)	0.0041	<0.0001
Asian	230 (1.74%)	483 (1.74%)	0.0018	<0.0001
African American	1,284 (9.74%)	2,697 (9.74%)	0.0744	<0.0001
Native Hawaiian or Other	18 (0.14%)	38 (0.14%)	0.0009	<0.0001
White	10,106 (76.62%)	21,229 (76.62%)	0.0517	<0.0001
Unknown	1,457 (11.05%)	3,061 (11.05%)	0.0159	<0.0001
**Ethnicity**				
Hispanic or Latino	1,002 (7.60%)	2,105 (7.60%)	0.0323	<0.0001
Not Hispanic or Latino	9,407 (71.32%)	19,760 (71.32%)	0.0072	<0.0001
Unknown	2,780 (21.08%)	5,840 (21.08%)	0.0251	<0.0001
Antibiotics used after discharge	2,440 (18.50%)	5,125 (18.50%)	0.1022	<0.0001
Nicotine use	1,226 (9.30%)	2,575 (9.30%)	0.0199	<0.0001
Risk Factors	224 (1.70%)	471 (1.70%)	0.0015	<0.0001
CNS Medications	11,130 (84.39%)	23,379 (84.39%)	0.4656	<0.0001
Hormones/Synthetics/Modifiers	8,901 (67.49%)	18,697 (67.49%)	0.3716	<0.0001
Diabetes	1,052 (7.98%)	2,210 (7.98%)	0.0017	<0.0001
Thyroid/Endocrine Issues	126 (0.96%)	265 (0.96%)	0.0006	<0.0001
Obesity	634 (4.81%)	1,332 (4.81%)	0.019	<0.0001
Other Metabolic Disorders	3,604 (27.33%)	7,571 (27.33%)	0.0791	<0.0001
Diseases of the Heart/Vasculature	3,080 (23.35%)	6,470 (23.35%)	0.0008	<0.0001
Respiratory Diseases/Illnesses	1,405 (10.65%)	2,951 (10.65%)	0.0236	<0.0001
MSK/Joint/Connective Tissue Diseases	2,746 (20.82%)	5,768 (20.82%)	0.0491	<0.0001
Other CNS-Related Issues	663 (5.03%)	1,393 (5.03%)	0.0004	<0.0001
Inflammation/Infection	7,798 (59.13%)	16,380 (59.12%)	0.3404	<0.0001
Pain	12,367 (93.77%)	25,978 (93.76%)	0.4565	<0.0001
Lack of Normal Physiological Development	8 (0.06%)	17 (0.06%)	0.0002	<0.0001
Malnutrition and Nutritional Deficiencies	127 (0.96%)	267 (0.96%)	0.0026	<0.0001
Factors Influencing Mental Health Status	864 (6.55%)	1,815 (6.55%)	0.0244	<0.0001
Healthcare Encounters	2,554 (19.36%)	5,365 (19.36%)	0.022	<0.0001
Lifestyle/Environmental Stressors	206 (1.56%)	433 (1.56%)	0.0026	<0.0001
Allergy to Antibiotic/Antiinfective Agents	139 (1.05%)	292 (1.05%)	0.0083	<0.0001

Absolute standardized mean differences (ASMDs) for all matching variables were calculated for the original data subset and the weighted data subset. ASMDs were computed after weighting by taking the mean for each variable among the treatment group and the mean for each variable among the control group. These values were subtracted and standardized by dividing the difference by the standard deviation before any adjustments. The goal of weighting was to make adjustments such that the sample mimics a randomized trial. Small ASMDs (typically ≤0.1) indicate that the weighting method has balanced the groups to resemble a randomized trial (Rubin, 2001). After weighting ASMDs for all of the covariates fell below the 0.1 benchmark (Table 1 and Table 2).

*Primary Analyses.* Separate weighted analyses were generated for each sex and age group using the entropy balancing algorithm. Univariate logistic regression models were used to estimate parameters for each weighted sex and age effect. The resultant parameter estimates from the separate entropy balanced analyses, along with their associated standard errors, were used to compare estimated exposure effects across groups using Wald test statistics.

## Results

### Participants

Dataset of included participants consisted of 61,769 individuals. This cohort was first stratified by age and then re-analyzed after stratification by age.

Total sample size of female patients was 36,718–11,620 antibiotic-treated and 25,098 control (Table 1). The mean age at hospitalization in the treatment group was 52.45 ± 17.40 years (mean ± SD) and 53.98 ± 16.76 years in the control group. Total sample size of male patients was 25,051–8,594 treated with antibiotics and 16,457 control (Table 1). The mean age at hospitalization for the antibiotic treatment group was 57.87 ± 15.02 years and 57.28 ± 15.46 years in the control group. The groups were well balanced with an ASMD <0.0001 after weighting. All other variables given in Table 1 can be assessed in a similar manner.

Patient characteristics for the three age groups used for entropy balancing are summarized in Table 2. The total sample size of patients in the 18–25-year-old bracket was 3,135–1,052 antibiotic-treated and 2,083 control. In this age group, the mean age at hospitalization was 22.3 ± 2.04 years and 22.21 ± 1.99 years in the treatment group and in the control group, respectively. The total sample size of patients in the 26–49-year-old bracket was 17,740–5,973 antibiotic-treated and 11,767 control. The mean age at hospitalization was 37.91 ± 6.92 and 38.69 ± 6.97 in the treatment group and in the control group, respectively. The total sample size of patients in the 50 and older bracket was 40,894–13,189 treated and 27,705 control. The mean age at hospitalization in the treatment group was 64.97 ± 9.26 and 64.82 ± 9.44 in the control group.

### Outcomes: Treatment group effects stratified by sex

**FIGURE 1:**
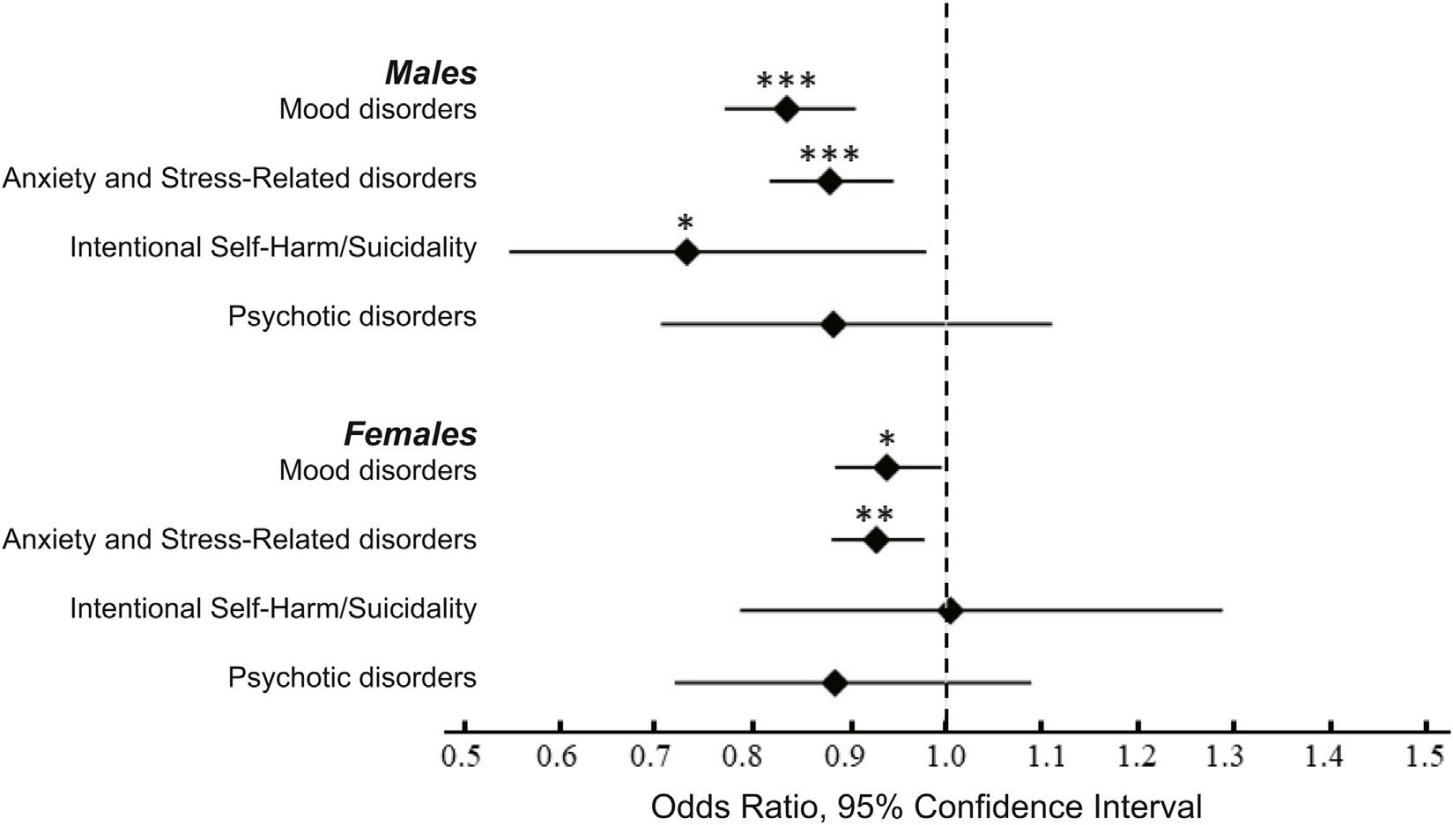
Odds ratios of for the emergence psychiatric conditions following inpatient antibiotic exposure. The data were analyzed after stratification by sex. Statistical analyses revealed a significant decrease in the risk of mood disorders and anxiety and stress-related disorders in men and women. We also observed a decrease in the risk of intentional self-harm/suicidality in males. For additional details see Table 3. * -- *p* < 0.05, ** -- *p* < 0.001, *** -- *p* < 0.0001.

Among women, we found significantly reduced risk of mood disorders (OR 0.94, 95% CI 0.89–1.00; *p* = 0.040) and anxiety and stress-related disorders (OR 0.93, 95% CI 0.88–0.98; *p* = 0.006; Table 3 and Figure 1). Among men, antibiotic use was associated with significantly reduced risk of: mood disorders (OR 0.84, 95% CI 0.77–0.91; *p* < 0.0001), anxiety and stress-related disorders (OR 0.88, 95% CI 0.82–0.95; *p* = 0.0006), and intentional self-harm and suicidality (OR 0.73, 95% CI 0.55–0.98; *p* = 0.040; Table 3 and Figure 1).

**TABLE 3:** Impact of antibiotic administration on psychiatric outcomes in female and male cohorts. NA–not applicable, OR–odds ratio.

	Exposed			Unexposed				
Outcome	Patients in group, No.	Patients with outcome, No.	Incidence rate	Patients in group, No.	Patients with outcome, No.	Incidence rate	OR (95% CI)	*p*-value
**Females (N = 36,718)**								
Total, No.	11,620	NA	NA	25,098	NA	NA		
Mood Disorders	NA	1,924	16.56%	NA	4,376	17.44%	**0.94 (0.89,1.00)**	**0.040**
Anxiety and Stress-Related Disorders	NA	2,592	22.31%	NA	5,925	23.61%	**0.93 (0.88, 0.98)**	**0.006**
Intentional Self-Harm and Suicidality	NA	93	0.80%	NA	200	0.80%	1.01 (0.79, 1.29)	0.96
Psychotic Disorders	NA	128	1.10%	NA	312	1.24%	0.89 (0.72, 1.09)	0.25
**Males (N = 25,051)**								
Total, No.	8,594	NA	NA	16,457	NA	NA		
Mood Disorders	NA	936	10.89%	NA	2,099	12.76%	**0.84 (0.77, 0.91)**	**<0.0001**
Anxiety and Stress-Related Disorders	NA	1,220	14.20%	NA	2,606	15.83%	**0.88 (0.82, 0.95)**	**0.0006**
Intentional Self-Harm and Suicidality	NA	63	0.73%	NA	164	1.00%	**0.73 (0.55, 0.98)**	**0.040**
Psychotic Disorders	NA	110	1.28%	NA	238	1.44%	0.88 (0.70, 1.11)	0.30

Bolded values indicate statistically significant differences at *p* < 0.05.

### Outcomes: Treatment group effects stratified by age

**FIGURE 2:**
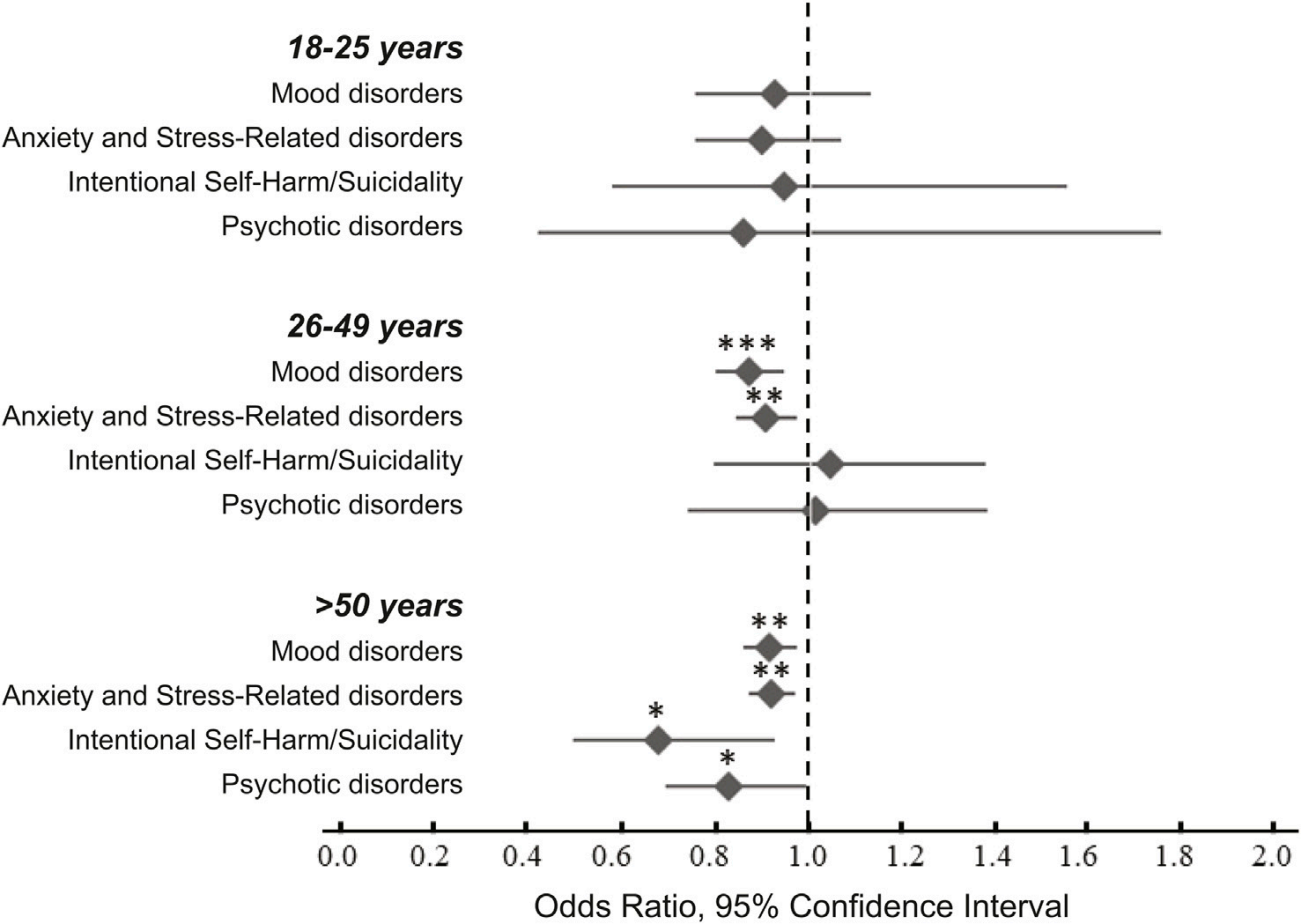
Odds ratios of for the emergence psychiatric conditions following antibiotic exposure during hospitalization. The data were analyzed after stratification by age. Statistical analyses revealed significant decreased risk of mood disorders and anxiety and stress-related disorders in the 26–49 years and in the ≥50 years age groups. There was also a significant decrease in the risk of intentional harm/suicidality and psychotic disorders in the ≥50 years group. For additional details see Table 4. * -- *p* < 0.05, ** -- *p* < 0.001, *** -- *p* < 0.0001.

We did not detect a significant relationship between antibiotic use and any of the outcome measures in the 18–25 years group. In the 26–49 years group we observed significant reduction in the risk of mood disorders (OR 0.87, 95% CI 0.80–0.94; *p* = 0.0009) and in that of anxiety and stress-related disorders (OR 0.90, 95% CI 0.84–0.97; *p* = 0.0072; Table 4 and in Figure 2). In the ≥50 years group we detected significant reductions in all four outcome variables: mood disorders (OR 0.91, 95% CI 0.86–0.97; *p* = 0.0031), anxiety and stress-related disorders (OR 0.92, 95% CI 0.87–0.97; *p* = 0.0018), intentional self-harm and suicidality (OR 0.67, 95% CI 0.49–0.92; *p* = 0.014), and psychotic disorders (OR 0.83, 95% CI 0.69–0.99; *p* = 0.040; Table 4 and in Figure 2).

**TABLE 4:** Impact of antibiotic administration in cohorts stratified by age. NA–not applicable, OR–odds ratio.

	Exposed			Unexposed				
Outcome	Patients in group, No.	Patients with outcome, No.	Incidence rate	Patients in group, No.	Patients with outcome, No.	Incidence rate	OR (95% CI)	*p*-value
**Age 18–25 years (N = 3,135)**								
Total, No.	1,052	NA	NA	2,083	NA	NA		
Mood Disorders	NA	166	15.78%	NA	351	16.85%	0.92 (0.75, 1.13)	0.44
Anxiety and Stress-Related Disorders	NA	250	23.76%	NA	538	25.83%	0.90 (0.75, 1.07)	0.22
Intentional Self-Harm and Suicidality	NA	24	2.28%	NA	50	2.40%	0.94 (0.58, 1.55)	0.82
Psychotic Disorders	NA	11	1.05%	NA	25	1.20%	0.86 (0.42, 1.75)	0.67
**Age 26–49 years (N = 17,740)**								
Total, No.	5,973	NA	NA	11,767	NA	NA		
Mood Disorders	NA	957	16.02%	NA	2,120	18.02%	0.87 (0.80, 0.94)	**0.0009**
Anxiety and Stress-Related Disorders	NA	1,361	22.79%	NA	2,896	24.61%	0.90 (0.84, 0.97)	**0.0072**
Intentional Self-Harm and Suicidality	NA	80	1.34%	NA	151	1.28%	1.04 (0.80, 1.37)	0.76
Psychotic Disorders	NA	61	1.02%	NA	119	1.01%	1.01 (0.74, 1.38)	0.95
**Age >50 years (N = 40,894)**								
Total, No.	13,189	NA	NA	27,705	NA	NA		
Mood Disorders	NA	1,737	13.17%	NA	3,949	14.25%	0.91 (0.86, 0.97)	**0.0031**
Anxiety and Stress-Related Disorders	NA	2,201	16.69%	NA	4,971	17.94%	0.92 (0.87, 0.97)	**0.0018**
Intentional Self-Harm and Suicidality	NA	52	0.39%	NA	162	0.58%	0.67 (0.49, 0.92)	**0.014**
Psychotic Disorders	NA	586	4.44%	NA	420	1.52%	0.83 (0.69, 0.99)	**0.040**

Bolded values indicate statistically significant differences at *p* < 0.05.

## Discussion

In the current study we utilized a large multi-site database to generate large well-balanced cohorts stratified by sex and by age. Our analyses indicate that antibiotic administration in the inpatient setting is associated with protective effects on psychiatric outcomes. Our most consistent observation was that antibiotic administration was associated with lower risk of mood disorders and anxiety and stress-related disorders. This was true in females, males, and in individuals 26–49 years old and in those ≥50 years old. Similarly, risk of self-harm and suicidality was lower in males and in individuals ≥50 years old. Subjects in this older age group were also at lower risk of psychosis, likely due to the improvement of delirium after treatment of infection (Rummans et al., 1995).

One of the strengths of our study is that we had access to a large multisite database that contained nearly 70 million patient records. Thus, were able to carefully balance cohorts and to control for a large number confounds, including as race, sex, age, medical comorbidities, and co-administration of CNS medications. We also utilized STROBE criteria to minimize bias in our study as well as to increase reliability (von Elm et al., 2007b; von Elm et al., 2007c; von Elm et al., 2007d). It is well established that minimization of bias is critically important in a variety of clinical studies, including retrospective cohort studies such as this one (von Elm et al., 2007b; von Elm et al., 2007c; von Elm et al., 2007d) as well as in meta-analyses and reviews (Morgan et al., 2018a; Morgan et al., 2019).

Previous reports found positive associations between antibiotic use and anxiety (Lurie et al., 2015), depression (Lurie et al., 2015), mania (Walrave et al., 2016; Yolken et al., 2016; Lambricht et al., 2017; Puri et al., 2021), suicidal ideation (LaSalvia et al., 2010; Kaur et al., 2016), and psychosis (Farrington et al., 1995; Moorthy et al., 2008; Michalak et al., 2017). However, much of this evidence is based on case reports, studies with limited numbers of subjects and without appropriate control for confounding variables.

Large-scale that explored the link between antibiotic exposure and psychiatric outcomes provide a mixed picture. Lurie, et al. reported an increase in the risk of depression and anxiety, but not of psychosis (Lurie et al., 2015), while Wilcox, et al. did not find any adverse effects as a consequence of antibiotic exposure (Wilcox et al., 2020). Others have reported a decrease in the risk of opioid use disorder (OUD) when opioids are co-prescribed with antibiotics (Freedman et al., 2022), while administration of antibiotics alone can increase risk of OUD (Clegg et al., 2023). Our observations extend these prior observations and indicate that antibiotic administration in hospitalized patients can decrease the risk of novel psychiatric disorders in a sex- and age-dependent manner. A significant strength of our approach is the use of entropy balancing to account for group differences in several factors that may alter risk for neuropsychiatric disorders. We controlled for multiple confounding variables, such as inflammation, CNS medications, pain, medical co-morbidities (e.g. obesity, cardiovascular disease, and diabetes), and others. We only considered patients who were administered antibiotics while in hospital, suggesting that type of setting in which these medications are prescribed may be an important determinant of their effects on mental health.

Our study is consistent with prior reports of protective effects of antibiotics in mental health. While traditionally considered to worsen depression and suicidality, a more recent analysis suggests that isotretinoin protects against several neuropsychiatric outcomes (Kridin and Ludwig, 2023). A similar study found no associated protective or harmful effect of isotretinoin on neuropsychiatric outcomes (Paljarvi et al., 2022). Minocycline adjunctive therapy may be beneficial in schizophrenia, with documented improvement in cognitive (Levkovitz et al., 2010; Zhang et al., 2019), positive (Zhang et al., 2019), and negative (Levkovitz et al., 2010; Liu et al., 2014; Palleja et al., 2018) symptoms and reduction of inflammatory cytokines IL-1β and IL-6 (Palleja et al., 2018; Zhang et al., 2019). Minocycline treatment may also have protective effects on depressive disorders (Cai et al., 2020; Husain et al., 2020). Taken together with our observations, these results suggest that antibiotic administration is protective against a range of neuropsychiatric outcomes.

Timing of antibiotic exposure along with classes of medications used may be critical factors in associated risk of mental illness. Children exposed to antibiotics in the first 3 years of life have increased risk for mood and anxiety disorders, depending on antibiotic class (Delara et al., 2020). Specifically, postnatal exposure to tetracyclines, aminoglycosides, quinolones, or sulfonamides was associated with increased risk of mood and anxiety disorders by the time study participants were adolescents (Delara et al., 2020). Conversely, exposure to macrolides, lincosamides, or streptogramins were associated with reduced risk of mood and anxiety disorders (Delara et al., 2020). Our study focused exclusively on late adolescent and adult exposure (18 + years of age at hospital admission). In general, younger adults (18–50 years) are at a greater risk for psychiatric disorders when compared to older populations (Bose et al., 2018). While antibiotic exposure had no effect on risk of novel psychiatric disorders in the youngest age group (18–25 years) in this study, we observed reduced risk of mood disorders and anxiety and stress-related disorders in the 26–49-year-old group, and decreased risk of mood disorders, anxiety and stress-related disorders, self-harm/suicidality, and psychotic disorders in the 50 years and older age group. Thus, protective effects of antibiotic exposure in hospitalized individuals may be age dependent.

### Limitations

There are several limitations to the current study due to the reliance on electronic healthcare records. We were unable to fully account for differences in socioeconomic status and environmental confounding factors between groups by using real-time EMR data (Morgan et al., 2018a; Morgan et al., 2018b; Morgan et al., 2019). We attempted to address this by balancing cohorts based on “Factors influencing contact with Health services (Z00-Z99)”. These codes include various items such as: body mass index (Z68), persons with potential health hazards related to socioeconomic and psychosocial circumstances (Z55-Z65), and a variety of reasons people may encounter health care services. Some of these shortcomings were mitigated by our use of the STROBE guideline and checklist to minimize bias and improve reliability (von Elm et al., 2007c; von Elm et al., 2007d).

This study was restricted to an inpatient population thus limiting the overall generalizability of results to the population at large. However, our study provides evidence that inpatient antibiotic administration may be beneficial to the individual’s long-term mental health. Prior studies that used electronic healthcare records may not have adequately balanced cohorts, or may not have covaried for all confounding variables, potentially resulting in erroneous conclusions.

Additionally, we were limited with regards to the antibiotic classes, duration of treatment, indications for antibiotic prescribing, and the impact of stratifying by these variables on psychiatric outcomes. Due to the nature of our de-identified real-time data, TriNetX relies on prescription claims to compile medication treatment information. Consequently, detailed information such as the duration of treatment and specific indications is not available within our dataset. We did not distinguish between different classes of antibiotics, and included patients that were treated with any class of these medications. Specific medications and antibiotic classes are listed in Supplemental Table S2. Future studies will be required to determine the impact of specific classes of antibiotics on psychiatric outcomes.

### Conclusions

We found that antibiotic treatment during hospitalization is associated with decreased subsequent risk of several classes of psychiatric disorders, which depended on the patients’ sex and age. Our findings suggest that antibiotic treatment in the hospital setting may provide protection against future psychiatric disorders. While antibiotic administration may be protective in the hospital setting, outpatient and/or off-label use of certain classes of antibiotics may predispose individuals for mood or anxiety disorders (Kaur et al., 2016). Future prospective studies should be able to parse out these differences.

## Data Availability

Publicly available datasets were analyzed in this study. This data can be found here: https://www.trinetx.com/. The raw data will be made available upon request.
